# Exosome-based detection of activating and resistance *EGFR* mutations from plasma of non-small cell lung cancer patients

**DOI:** 10.18632/oncotarget.26885

**Published:** 2019-04-23

**Authors:** Elena Castellanos-Rizaldos, Xuan Zhang, Vasisht R. Tadigotla, Dominik G. Grimm, Chris Karlovich, Luis E. Raez, Johan K. Skog

**Affiliations:** ^1^ Exosome Diagnostics, a Bio-Techne brand, Waltham, Massachusetts, USA; ^2^ Exosome Diagnostics, a Bio-Techne brand, Martinsried, Germany; ^3^ Leidos Biomedical Research Inc., Frederick National Laboratory for Cancer Research, Frederick, MD, USA; ^4^ Memorial Cancer Institute, Memorial Health Care System, Florida International University, Florida, USA

**Keywords:** liquid biopsy, exosomes, ctDNA, exoNA, NSCLC

## Abstract

Non-small cell lung cancer (NSCLC) is the most prevalent form of lung cancer and its molecular landscape has been extensively studied. The most common genetic alterations in NSCLC are mutations within the epidermal growth factor receptor (*EGFR*) gene, with frequencies between 10-40%. There are several molecular targeted therapies for patients harboring these mutations.

Liquid biopsies constitute a flexible approach to monitor these mutations in real time as opposed to tissue biopsies that represent a single snap-shot in time. However, interrogating cell free DNA (cfDNA) has inherent biological limitations, especially at early or localized disease stages, where there is not enough tumor material released into the patient’s circulation.

We developed a qPCR- based test (ExoDx *EGFR*) that interrogates mutations within *EGFR* using Exosomal RNA/DNA and cfDNA (ExoNA) derived from plasma in a cohort of 110 NSCLC patients.

The performance of the assay yielded an overall sensitivity of 90% for L858R, 83% for T790M and 73% for exon 19 indels with specificities of 100%, 100%, and 96% respectively. In a subcohort of patients with extrathoracic disease (M1b and MX) the sensitivities were 92% (L858R), 95% (T790M), and 86% (exon 19 indels) with specificity of 100%, 100% and 94% respectively.

## INTRODUCTION

Lung cancer represents one of every five cases of cancer-related death worldwide [1]. Non-small cell lung cancer (NSCLC) is one of the most common types of lung cancer and is divided into three main histological sub-types: squamous cell carcinoma, large cell carcinoma, and adenocarcinoma. NSCLC is very heterogeneous at the molecular level and between 10-40% of patients’ tumors harbor mutations within the epidermal growth factor receptor (*EGFR*) [2, 3].

There are now several molecular targeted therapies approved for this group of patients. Included in this group are the anti- EGFR first- and second-generation tyrosine kinase inhibitors (TKIs), which bind reversibly and irreversibly to the tyrosine kinase receptor. These are classified as evidence-based first-line treatments for NSCLC patients that harbor activating *EGFR* mutations (within exon 19 and the L858R missense mutation within exon 21) [4]. However, during treatment with these 1st- and 2nd- generation TKIs, there is an acquired resistance mechanism that arises through a missense driver mutation within exon 20 of *EGFR* (T790M) [5, 6]. This has led to the development and approval of a third-generation inhibitor, osimertinib, an oral, irreversible EGFR-TKI that is selective for both activating and resistance mutations [7].

Given the availability of these targeted therapies and higher survival rates of patients with early stage disease [8], it is critical to be able to monitor the tumor mutation profile with a sensitive and specific assay as early as possible [9].

The field of liquid biopsies has evolved greatly, and there are now several studies showing that longitudinal monitoring of cfDNA can capture tumor dynamics in NSCLC patients [10–13]. However, liquid biopsies that utilize the cell-free DNA fraction (cfDNA) face biological challenges. The majority of cfDNA is from normal cells and the level of cell-free tumor DNA (ctDNA) in that fraction can sometimes be below the limit of detection even with the most sensitive assay platforms [14, 15]. To address this limitation, three recent studies showed the benefits of combining the mutations found in exosomal nucleic acids with cfDNA (exoNA) for mutation detection [16, 17]. In one of the studies a CLIA validated qPCR test for *EGFR* T790M mutations in NSCLC patients (*n =* 210) achieved a clinical sensitivity and specificity of 92% and 89%, respectively [18].

**Figure 1:**
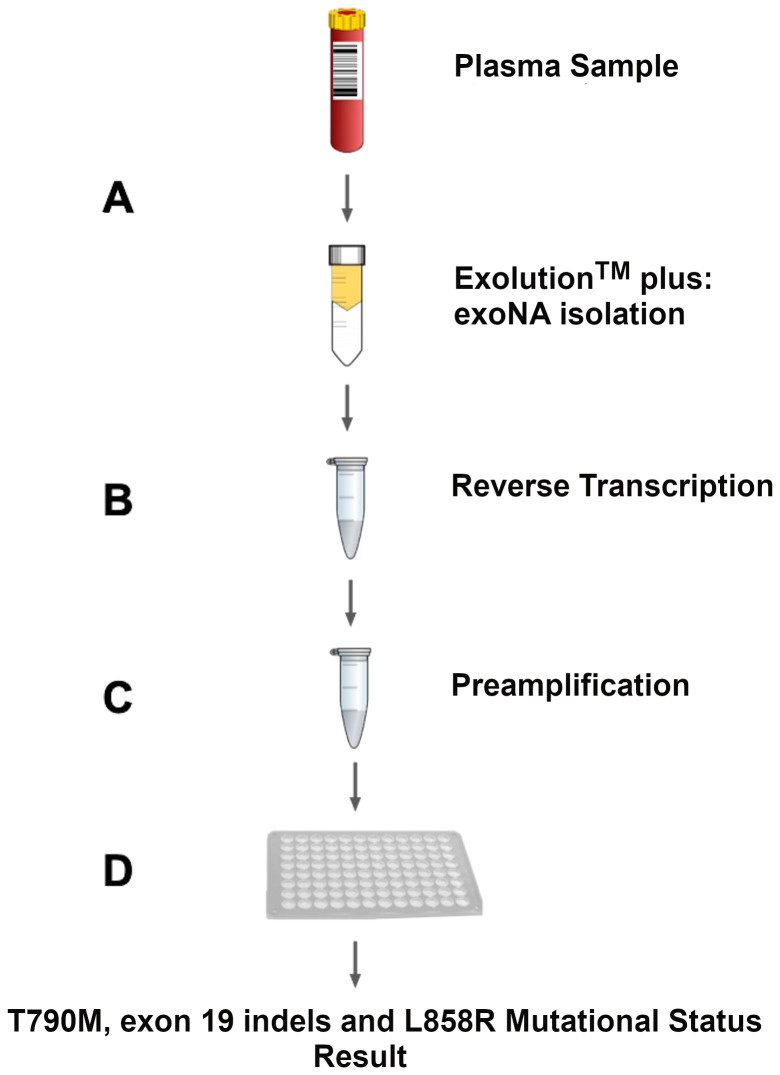
Assay workflow overview. (**A**) Column-based exosomal RNA/DNA and cfDNA (exoNA) isolation from plasma of NSCLC patients. (**B**) Reverse transcription step. At this step, we added an exogenous RNA construct as control to monitor for PCR inhibition to each sample. (**C**) Pre-amplification step of exon 19, 20 and 21, in addition to QBeta and exon 7 (controls for sample inhibition and integrity, respectively). (**D**) Amplification Refractory Mutation System (ARMS) based quantitative PCR step.

The goal of this study was to develop a qPCR-based test that interrogates a panel of 29 mutations in the *EGFR* gene, including the activating and resistance mutations (Table 1) that predict response to first line EGFR inhibitors and osimertinib. To mitigate the biological limitation of cfDNA assays we used the same approach as previously [16-18] and developed the assay on a combination of exosomal RNA/DNA and cfDNA (Figure 1).

**Table 1:** List of single point mutations, insertions and deletions interrogated with the ExoDx *EGFR* assay

Variant name	Variant type	Exon location in *EGFR*	Cosmic ID
c.2235_2249del15	15 bp deletion	Exon 19	COSM6223
c.2235_2248>AATTC	5 bp insertion		COSM13550
c.2235_2251>AATTC			COSM13552
c.2235_2252>AAT	15 bp deletion		COSM13551
c.2236_2250del15			COSM6225
c.2236_2253del18	18 bp deletion		COSM12728
c.2236_2253del18	5 bp insertion		COSM12416
c.2237_2253>TTGCT			COSM12367
c.2237_2254del18	1 bp insertion		COSM12384
c.2237_2255>T	3 bp insertion		COSM18427
c.2238_2248>GC	2 bp insertion		COSM12422
c.2238_2248>GC	3 bp insertion		COSM12419
c.2238_2252del15	15 bp deletion		COSM23571
c.2238_2255del18	18 bp deletion		COSM6220
c.2239_2247delTTAAGAGAA	9 bp deletion		COSM6218
c.2239_2248TTAAGAGAAG>C	10 bp insertion		COSM12382
c.2239_2251>C	1 bp insertion		COSM12383
c.2239_2253del15	15 bp deletion		COSM6254
c.2237_2253>TTGCT	3 bp insertion		COSM12403
c.2239_2256del18	18 bp deletion		COSM6255
c.2239_2258>CA	2 bp insertion		COSM12387
c.2240_2251del12	12 bp deletion		COSM6210
c.2240_2254del15	15 bp deletion		COSM12369
c.2240_2257del18	18 bp deletion		COSM12370
2235_2255>AAT	3 bp insertion		COSM12385
c.2237_2251del15	15 bp deletion		COSM12678
c.2237_2252>T	1 bp insertion		COSM12386
T790M	Missense mutation	Exon 20	COSM481727
L858R	Missense mutation	Exon 21	COSM6224

## RESULTS

### Analytical performance

The analytical sensitivity was evaluated by spiking synthetic constructs into 2 mL of healthy pooled plasma to generate a complex background and simulate a biological sample. The results for T790M in this new test were in alignment with our previous study [18]. As shown in Table 2A, 1.25 copies of T790M/mL of plasma were detected 75% of the time (3/4 biological replicates). At ≥ 5 copies/mL, we detected T790M in all biological replicates.

**Table 2:** Evaluation of ExoDx *EGFR* on T790M (A), L858R (B) and exon 19 deletion (Δ746-750) (C)

(A)								
Copies of *EGFR*/mL	4416 (Stdev±14.4)							
Copies T790M /mL	0	1.25	4	4.4	8	13	27	Total
**Fractional abundance (%)/mL**	0	0.03	0.09	0.10	0.18	0.30	0.61	
**Detected**	0	3	3	4	4	4	4	**22**
**Not detected**	4	1	1	0	0	0	0	**6**
Hit Fraction^*^	0	0.75	0.75	1.0	1.0	1.0	1.0	

*Hit fraction: Samples correctly classified.

**Table:** 

(B)											
Copies of *EGFR*/mL	4416 (Stdev±14.4)										
Copies L858R /mL	0	1.9	2.4	3.5	5.4	9.5	10.2	10.8	24.1	56	Total
**Fractional abundance (%)/mL**	0	0.04	0.05	0.08	0.12	0.21	0.23	0.24	0.54	1.25	
**Detected**	0	5	5	5	5	5	5	5	5	5	**45**
**Not detected**	5	0	0	0	0	0	0	0	0	0	**5**
Hit Fraction^*^	0	1.0	1.0	1.0	1.0	1.0	1.0	1.0	1.0	1.0	

**Table:** 

(C)											
Copies of *EGFR*/mL	4416 (Stdev±14.4)										
Copies exon 19 deletion Δ746-750 /mL	0	2	2.9	5.4	5.2	7.1	8.6	12.8	27.9	50	Total
**Fractional abundance (%)/mL**	0	0.05	0.07	0.12	0.12	0.16	0.19	0.29	0.63	1.12	
**Detected**	0	5	4	5	5	5	5	5	5	5	**45**
**Not detected**	5	0	1	0	0	0	0	0	0	0	**5**
Hit Fraction^*^	0	1.0	0.75	1.0	1.0	1.0	1.0	1.0	1.0	1.0	

Analytical performance was assessed by spiking mutations into healthy pooled plasma. Spike-ins were done in 2 mL plasma aliquots, but copies are depicted as copies/mL.

Table 2B and 2C summarize the assay results on activating mutations. The assay detected all five replicates at 2 copies of L858R/mL of plasma (equivalent to 0.04% allelic frequency). For exon 19 indels, 2 copies/mL (0.05% allelic frequency) were detected in all five replicates and 3 copies/mL were detected in 4/5 replicates (0.07% allelic frequency). The allelic frequencies were validated using droplet digital PCR (ddPCR). The number of mutant molecules in a plasma sample vary according to a Poisson distribution and at very low copy numbers, the variation across replicates is dominated by sampling noise rather than qPCR variation [19]. Note that the lowest theoretical limit of detection for PCR is 3 copies (assuming a Poisson distribution, 95% chance of including at least 1 copy in the PCR and single copy detection) under this assumption due to stochastic variation [20].

**Figure 2:**
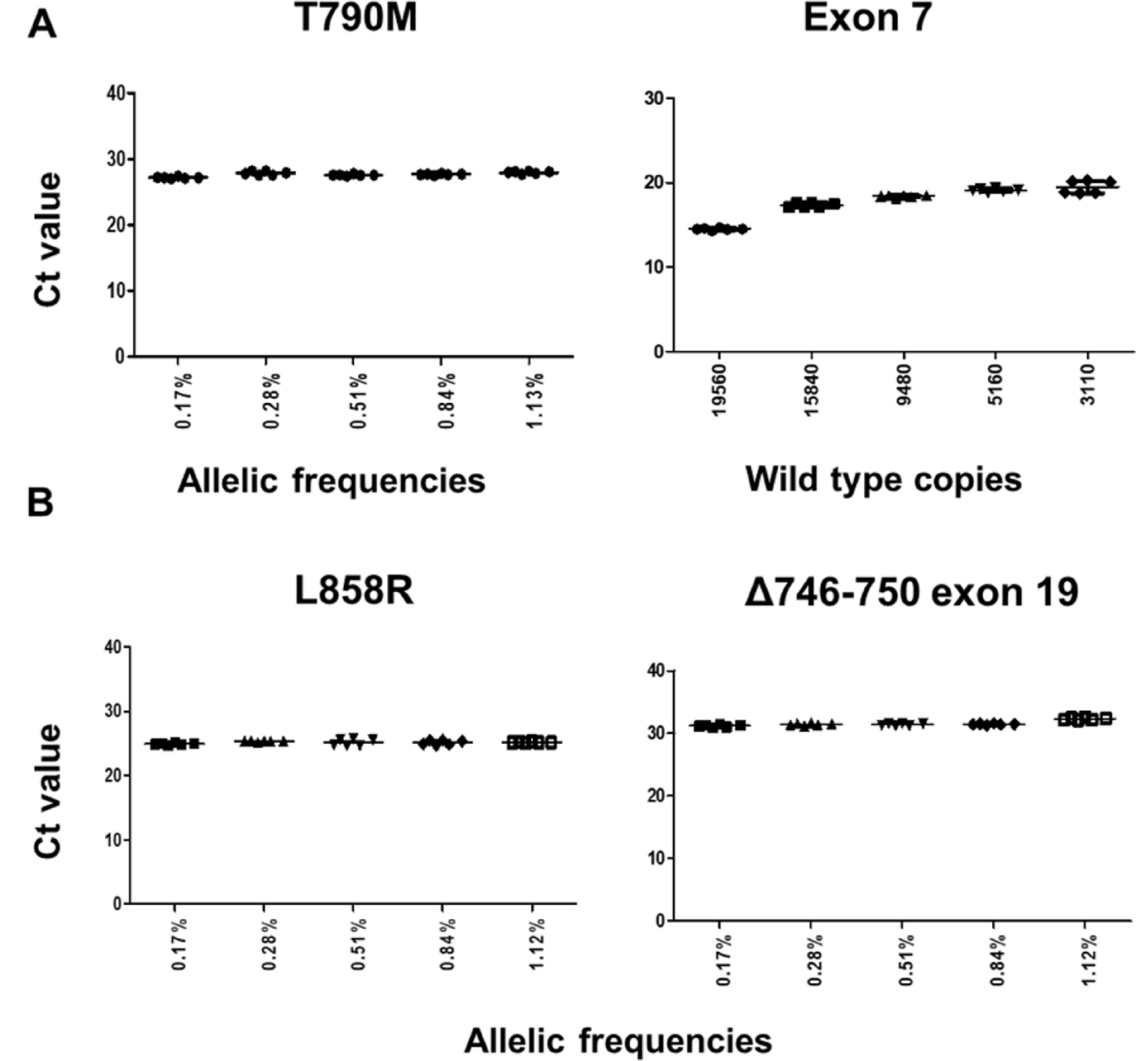
Assay robustness on gDNA admixtures for T790M, L858R and exon 19 deletion (Δ746-750). An average of 30 copies of mutant-containing synthetic DNA was used for the blends. (**A**) CT values for the T790M ARMS assay across different gDNA blends. (left). CT values for the *EGFR* exon 7 (control assay) (right). (**B**) CT values for the L858R ARMS assay (left). CT values for the exon 19 indels ARMS assay (right).

Next, we assessed the robustness of the test on mutation blends containing an average of 30 copies of mutant synthetic constructs (T790M, L858R or the most prevalent exon 19 deletion (Δ746-750) synthesized by IDT Corporation, IL, USA into wild type genomic DNA (Catalog no. G147A, Promega Corporation, WI, USA). Final allelic frequencies (Supplementary Table 1) and mutant copies in the admixtures were measured by droplet digital PCR (ddPCR) (Supplementary Table 2). The qPCR assays for all three targets were robust, mutant-specific and unaffected by the presence of wild type background (Figure 2), as we did not observe a CT delay in any of the allelic frequencies (0.17–1.13%) evaluated in a total of 35 samples, regardless of the wild type background.

We also estimated how the assays performed on a series of commercially available cell free DNA Reference Standards (Catalog nos. HD777, HD778 and HD779, Horizon Discovery, UK). These standards contain variable amounts of mutant templates down to single copies for all three targets in the 0.1% admixture. The correlation coefficient for all three assays were greater than 0.99 on the different reference admixtures (Supplementary Figure 1).

### *EGFR* mutation detection using exosomal nucleic acids and cfDNA on clinical samples

This patient cohort consisted of 110 clinical samples with 60 mutation-positive NSCLC patients (L858R, exon 19 indels and T790M) and 50 mutation-negative NSCLC patients and healthy donor samples with no history of familial lung cancer. 30% of NSCLC patients were diagnosed with intrathoracic disease (M0/M1a).

**Figure 3:**
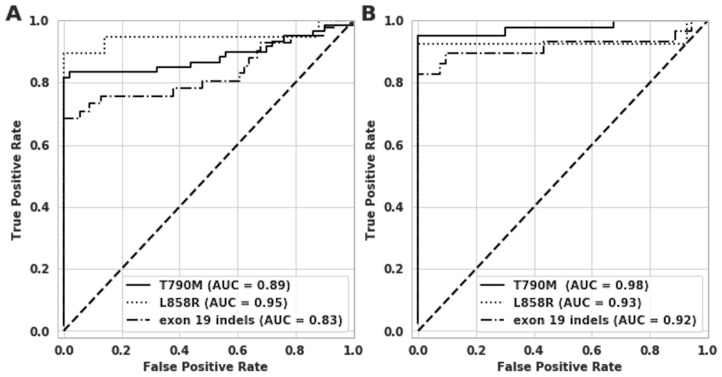
Receiver operating characteristic (ROC) curve analysis on clinical samples for T790M, L858R and exon 19 indels. The x-axes show 1-Specificity or the False Positive Rate (FPR), and y-axes show the sensitivity or True Positive Rate (TPR). (**A**) ROC curves for all three assays for all 110 patient samples. (**B**) ROC curves for all three targets on M1b/MX patient samples alone.

The clinical sensitivity of the assay was 83% for T790M, 90% for L858R and 73% for exon 19 indels with specificities of 100%, 100% and 96% respectively (Table 3A and 3B). The Area Under the Receiver Operator Characteristics Curve (ROC) for the T790M, L858R and exon 19 indels assays was 0.89, 0.95 and 0.83 respectively (Figure 3).

**Table 3:** Clinical cohort performance

A								
	Plasma results using exoNA							
		T790M		L858R^*^		exon 19 indels^*^		Total number
**Tissue result**		**+**	**-**	**+**	**-**	**+**	**-**	
	**+**	50	10	17	2	30	11	110
	**-**	0	50	0	91	3	66	

^*^Samples are mutually exclusive for L858R and exon 19 indels

**Table:** 

B						
Parameter	Clinical Samples					
	T790M		L858R		exon 19 indels	
	All^*^	M1b	All	M1b	All	M1b
**AUC**	0.89	0.98	0.95	0.93	0.83	0.92
**Specificity**	100.00	100.00	100.00	100.00	95.65	94.33
**Sensitivity**	83.33	95.24	89.47	92.31	73.17	86.21
**Accuracy**	90.91	97.56	98.18	98.78	87.27	91.46
**Precision**	100.00	100.00	100.00	100.00	90.91	89.29
**NPV**	83.33	95.24	97.85	98.57	85.71	92.59

^*^Overall cohort includes 30% of patients with intrathoracic disease.

(A) Confusion matrix that correlates tissue results obtained in tissue with plasma exoNA for the activating and resistance mutations for all patients included in the study. (B) Assay results for the activating and resistance mutations for the entire cohort (M1b/MX/M1a/M0) and patients with disease stages M1b/MX.

The assay also demonstrated very high performance among patient samples at disease stage M1b/MX with sensitivities of 95%, 92% and 86% for T790M, L858R and exon 19 indels respectively (Table 3B). Supplementary Table 3 lists the clinical samples that were negative in plasma for at least one *EGFR* mutation that were classified as positive in tissue, and hence likely false negative as classified by the assay. A majority of the false negative patient samples (9/15) had intrathoracic disease (M0/M1a). ~67% of the false negative M0/M1a samples were called negative for all of *EGFR* mutations (L858R, exon 19 indels and T790M) tested in this assay.

## DISCUSSION

The NSCLC field have been one of the pioneers for molecular therapies and personalized therapy based on individual tumor mutation status. For instance, nine superiority trials have proven the efficacy of several reversible and irreversible EGFR-TKIs versus chemotherapy alone in tumors with specific somatic variants (exon 19 indels and L858R in exon 21) [21]. Although these tumors have very good initial response rates, they eventually develop drug resistance via different mechanisms [22]. The most common mechanism of resistance is the missense mutation in codon 790 that results in an amino acid change (T790M) in exon 20 of *EGFR* [23]. A randomized, international, open-label, phase 3 clinical trial of osimertinib in 419 patients harboring this mutation showed a Progression Free Survival (PFS) of 10.1 months (95% CI: 8.3, 12.3) compared to 4.4 months (95% CI: 4.2, 5.6) for patients receiving doublet chemotherapy [24]. This resulted in the FDA approval of osimertinib for NSCLC patients with T790M-positive tumors.

This emphasizes the importance of assessing tumor mutational status using very accurate and sensitive methods not just prior to treatment but to track tumor dynamics over the course of therapy. In this arena, liquid biopsies have shown great potential as a tool for the clinical management of patients with NSCLC [25, 26]. However, sensitivity of liquid biopsies is limited by the amount of tumor-derived nucleic acids in circulation, especially in early stage disease. A study examining the circulating tumor DNA (ctDNA) fraction obtained from 640 subjects demonstrated that ctDNA fraction is highly variable, not only between tumor types but also within patients with the same tumor type. Only 55% of the patients with localized disease had detectable levels of ctDNA using 5 mL of plasma [27]. Sacher and collaborators recently published a prospectively validated plasma genotyping method with an overall sensitivity below 60% for patients with one metastatic site [28].

It is well known that cfDNA is not the only source of nucleic acids in biofluids but that extracellular vesicles such as exosomes also contain nucleic acids. cfDNA is typically described as coming from the dying process of the tumor (apoptosis and necrosis) whereas exosomes are released as an active metabolic process from living cells [29]. Therefore, combining these two approaches may overcome some of the inherent biological limitations of an approach based on cfDNA alone as well as assess mutation status from the living as well as the dying processes within the tumors. A recent study that compared the mutation detection rates with different liquid biopsy platforms showed a significantly higher performance when looking in the exoNA fraction compared to just cfDNA [16]. The largest added value with the exoNA over cfDNA was for patients with intrathoracic disease (M0/M1a), which is not surprising since many studies have shown limited ctDNA copies in this population. We have recently demonstrated that the exoNA approach is feasible for detection of the *EGFR* T790M mutation and yielded very high clinical performance (92% sensitivity and 89% specificity) for the detection of T790M in plasma of NSCLC patients [18]. The test was validated on 210 clinical samples, from which ~40% had localized disease.

There is a need for a broader *EGFR* panel beyond looking at the *EGFR* T790M mutation, so this study summarizes the results of a qPCR-based *EGFR* mutation panel that also includes the activating *EGFR* mutations. The assay had a sensitivity of 95% for T790M and 88% for activating mutations in the M1b/MX population, similar to what was achieved with BEAMing (Beads, Emulsion, Amplification, Magnetics) (95% T790M, 96% activating) and cobas® plasma test (95% T790M, 90% activating) [14]. However, the assay has a sensitivity of 56% for both T790M and activating mutations in patients with intrathoracic disease (M0/M1a) compared to 27% and 39% for BEAMing and 14% and 42% for the cobas® plasma test [14]. These results further support an increase in sensitivity that can be achieved by using exoNA in comparison to liquid biopsy tests that use only cfDNA, especially in patients with early stage disease. The increase in sensitivity does not adversely affect the specificity of the assay as demonstrated by the high specificities of 100%, 100% and 94% for T790M, L858R and exon 19 indels respectively (Table 3B). We also note that none of the *EGFR* mutations tested (L858R, exon 19 indels and T790M) were detected in majority of the false negative M0/M1 samples (Supplementary Table 3). The patients that remain problematic to detect with this improved liquid biopsy is likely due to the biology of the disease, releasing very low amounts of both cfDNA and exosomal RNA/DNA into circulation. A potential workaround could be to use larger plasma volumes (> 2 mL) to minimize the effects of sampling noise and improve the sensitivity of the assay. The exoNA extraction efficiency is linear, and roughly twice as much material is retained into the assay if the input volumes double.

The high sensitivity and specificity of this assay demonstrate its utility as a tool for the detection and monitoring of *EGFR* mutations in NSCLC patients to inform clinical management, especially in cases where tissue biopsies are not readily available.

## MATERIALS AND METHODS

### Assay design

The workflow for ExoDx (*EGFR*) is schematically represented in Figure 1. exoNA was extracted from plasma samples using a cGMP manufactured isolation kit (ExoLution^TM^ Plus, Exosome Diagnostics, Inc. Waltham, MA, USA) [16, 18]. ExoLution™ Plus uses a spin-column to capture both the cfDNA and extracellular vesicles. We excluded any vesicle larger than 0.8 μm in diameter from the plasma through a pre-filtration step [30]. The entire eluate was then reverse transcribed as described elsewhere [16, 30], followed by a pre-amplification reaction. This step contains *EGFR* exon 19 and 20 wild-type blockers as well as primers targeting *EGFR* exon 19, 20 and 21, exon 7 (control reaction) and a non-human control sequence (QBeta) that is spiked into every patient sample as a qPCR/inhibition control.

The final downstream detection step includes a quantitative PCR (qPCR) step using an Amplification-Refractory Mutation system (ARMS) approach to selectively amplify T790M, L858R and 27 different exon 19 insertion/deletions (indels) (Table 1). The assay includes controls for sample integrity and inhibition (*EGFR* exon 7 and QBeta) in each qPCR reaction as previously described [18].

### Determination of qPCR CT thresholds for *EGFR* L858R and exon 19 indels assays

We estimated the optimal cycle thresholds (CT) for L858R and exon 19 indels detection in plasma with synthetic construct spike-ins (Table 2, Supplementary Table 4). The CT threshold for T790M was derived previously [18]. The qPCR thresholds for each assay were estimated by maximizing the Youden’s J statistic [31], which simultaneously optimizes both sensitivity and specificity.

### Analytical Evaluation of ExoDx *EGFR*

The assay was first evaluated by spiking double-stranded synthetic DNA constructs (custom order, Integrated DNA Technologies, Coralville, IA, USA) containing T790M, L858R or exon 19 deletion Δ746-750 (c.2235_2249) into healthy pooled plasma obtained from 10 males and 10 female donors (Bioreclamation IVT, NY, USA) to simulate a patient plasma sample. The reference construct copy number determination was done by OD260 measurement from the manufacturer, and before each experiment, we performed an orthogonal PCR verification of the amplifiable copies of the reference constructs using the QX200 Droplet Digital PCR (ddPCR) System (BioRad, CA, USA) and commercially available assays (dHsaCP2000019, dHsaCP2000021 and dHsaCP2000039) following the manufacturer’s recommendations. A summary of reaction composition as well as cycling conditions can be found in Supplementary Table 5.

First, we assessed equivalency with the previously validated assay [18] to ensure that the addition of the activating mutations (L858R and exon 19 indels) did not negatively impact the assay. To do this, we tested 28 data points (four biological replicates/measurement) around the previously defined Limit of Detection (LoD). Evaluation was done using the same final assay cut-offs, QC metrics, synthetic templates and plasma samples [18] (Table 2).

The analytical conditions (thresholds) for L858R and the exon 19 deletion Δ746-750 was determined with the synthetic constructs (mutation containing sequences) spiked into healthy pooled plasma samples. The levels of *EGFR* in the plasma used for these experiments were also quantified by ddPCR to calculate allelic frequencies for the spike-in experiments. Allelic frequencies as low as 0.03%, 0.04% and 0.05% (estimated from ddPCR experiments) were tested for T790M, L858R and exon 19 deletion, respectively (Table 2).

The copy numbers used for the spike-in experiments for all three targets are summarized in Table 2. Input material used for these experiments were simultaneously measured by ddPCR using the commercial assays described above. Measured ddPCR copies values were used for these spike-in experiments (Supplementary Figure 2).

Next, in addition to the spike-in into plasma exoNA described above, the assay robustness for these three mutations was also assessed on a genomic DNA (gDNA) admixture. Wild-type Promega genomic DNA (125 ng/μl (37,879 genomic equivalents) (Catalog no. G147A, Promega Corporation, WI, USA), was mixed at different ratios keeping an average of 30 copies of mutant-containing DNA constant (previously quantified by ddPCR as described above) (Figure 2 and Supplementary Tables 1 and 2). The wild type gDNA background was also measured using the *EGFR* exon 7 assay (Figure 2).

### Evaluation of ExoDx *EGF*R on clinical samples

The accuracy of the assay was assessed on 110 clinical samples (Table 3), see Supplementary Table 6 for the clinical description. The NSCLC patient cohort consisted of 60 mutation-positive NSCLC patients (L858R, exon 19 indels and T790M) confirmed positive by tissue biopsy using institutionally approved methods and 50 mutation-negative NSCLC patients and healthy donor samples with no history of familial lung cancer. The median plasma input volume for the study was 2 mL (standard deviation of 0.5 mL).

The 60 mutation-positive NSCLC patients included in the study represented a variety of disease stages, with M0 (*n =* 5), M1a (*n =* 13), M1b (*n =* 39) and MX (*n =* 3). All mutation-positive patients had received prior treatment with at least one first generation TKI. The 50 negative samples included in the study were from NSCLC patients with confirmed-negative biopsy (*n* = 25) or healthy donors and pools with no history of cancer (*N* = 25). Samples were provided by Clovis Oncology, Inc. (Boulder, CO, USA), ALCMI (Wilmington, NC, USA), Memorial Cancer Institute (Hollywood, FL, USA), Althia Health, S.L (Barcelona, Spain) and Biopartners, Inc. (California, USA). Mutational status from all the NSCLC patient samples included in the study was independently assessed by institutionally-approved methods. All samples were collected under clinical study protocols approved for this purpose by their respective Institutional Review Boards (IRB).

## SUPPLEMENTARY MATERIALS





## Abbreviations

cfDNA: cell free DNA
ctDNA: circulating tumor DNA
exoNA: exosomal nucleic acids (DNA and RNA) and cfDNA
NSCLC: non-small cell lung cancer
*EGFR*: epidermal growth factor receptor
TKIs: tyrosine kinase inhibitors
ddPCR: droplet digital PCR
ARMS: Amplification refractory mutation system
gDNA: genomic DNA
