# Supplementary material for: Exosome-based detection of activating and resistance EGFR mutations from plasma of non-small cell lung cancer patients
Source: Oncotarget. 2019 Apr 23;10(30):2911–20. doi: 10.18632/oncotarget.26885 (PMC6499599; doi:10.18632/oncotarget.26885)
Supplement: Supplementary file 1 [file oncotarget-10-2911-s001.pdf]

Exosome-based detection of activating and resistance EGFR mutations from plasma of non-small cell lung cancer patients

SUPPLEMENTARY MATERIALS

Supplementary Table 1: Mutant admixtures

T790M		L858R		exon 19 indels		Mutation detected
Allelic frequency	Mutant copies*	Allelic frequency	Mutant copies*	Allelic frequency	Mutant copies*	
0% (Wild type)	0	0% (Wild type)	0	0% (Wild type)	0	No
0% (Wild type)	0	0% (Wild type)	0	0% (Wild type)	0	No
0% (Wild type)	0	0% (Wild type)	0	0% (Wild type)	0	No
0% (Wild type)	0	0% (Wild type)	0	0% (Wild type)	0	No
0% (Wild type)	0	0% (Wild type)	0	0% (Wild type)	0	No
0% (Wild type)	0	0% (Wild type)	0	0% (Wild type)	0	No
0.17%		0.17%		0.17%		Yes
0.17%		0.17%		0.17%		Yes
0.17%		0.17%		0.17%		Yes
0.17%		0.17%		0.17%		Yes
0.17%		0.17%		0.17%		Yes
0.17%		0.17%		0.17%		Yes
0.28%		0.28%		0.28%		Yes
0.28%		0.28%		0.28%		Yes
0.28%		0.28%		0.28%		Yes
0.28%		0.28%		0.28%		Yes
0.28%		0.28%		0.28%		Yes
0.28%		0.28%		0.28%		Yes
0.51%		0.51%		0.51%		Yes
0.51%		0.51%		0.51%		Yes
0.51%	26.1 ± 5.6	0.51%	31.5 ± 2.8	0.51%	32.5 ± 7.4	Yes
0.51%		0.51%		0.51%		Yes
0.51%		0.51%		0.51%		Yes
0.51%		0.51%		0.51%		Yes
0.84%		0.84%		0.84%		Yes
0.84%		0.84%		0.84%		Yes
0.84%		0.84%		0.84%		Yes
0.84%		0.84%		0.84%		Yes
0.84%		0.84%		0.84%		Yes
0.84%		0.84%		0.84%		Yes
1.13%		1.12%		1.12%		Yes
1.13%		1.12%		1.12%		Yes
1.13%		1.12%		1.12%		Yes
1.13%		1.12%		1.12%		Yes
1.13%		1.12%		1.12%		Yes
1.13%		1.12%		1.12%		Yes

*Average mutant copies measured by ddPCR. Average of six technical replicates.

Supplementary Table 2: Droplet digital PCR validation of the sample input for the allelic frequency experiments

Sample*	Copies Per 20 uL Well	Average**	Stdev	Sample*	Copies Per 20uL Well	Average**	Stdev	Sample*	Copies Per 20uL Well	Average**	Stdev
25 copies T790M	24.00	26.30	5.57	25 copies L858R	32.00	31.47	2.84	25 copies exon 19 deletion (Δ746-750)	19.40	32.53	7.45
	25.00				31.80				28.40		
	23.80				28.00				44.00		
	18.80				35.00				34.80		
	29.60				27.60				33.80		
	36.60				34.40				34.80		

*Estimated copies based on Optical Density (OD) measurements from commercial vendor; **Average of six technical ddPCR replicates.

Supplementary Table 3: Samples that have one or more false negative classification in plasma as compared with tumor

Patient no.	Tissue status			ExoNA results from plasma	M status
	T790M	L858R	exon 19 indels		
57	Positive	Negative	Positive	Negative for all	M1a
61	Positive	Negative	Positive	Negative for all	M0
62	Positive	Negative	Positive	Positive for T790M only	M1b
66	Positive	Negative	Positive	Positive for exon 19 indels only	M1b
68	Positive	Negative	Positive	Negative for all	M1a
72	Positive	Positive	Negative	Negative for all	M1a
73	Positive	Negative	Positive	Negative for all	M1a
75	Positive	Negative	Positive	Negative for all	M1a
83	Positive	Negative	Positive	Positive for exon19 indels only	M0
85	Positive	Positive	Negative	Negative for all	M1b
92	Positive	Negative	Positive	Positive for T790M only	M0
97	Positive	Positive	Negative	Positive for L858R only	M0
99	Positive	Negative	Positive	Positive for T790M only	M1b
100	Positive	Negative	Positive	Positive for T790M only	M1b
104	Positive	Negative	Positive	Positive for T790M only	M1b

Supplementary Table 4: Synthetic constructs spike-ins used in the study

Target	Sequence
T790M	ccctccctccagGAAGCCTACGTGATGGCCAGCGTGGACAACCCCCACGTGTGCCGCCTGCTGGGCATCTGCCTCACCTCCACCGTGCAGCTCATCATGCAGCTCATGCCCTTCGGCTGCCTCCTGGACTATGTCCGGGAACACAAAGACAATATTGGCTCCCAGTACCTGCTCAACTGGTGTGTGCAGATCGCAAAGgtaAtcagggaagggagatacggggaggggagataaggagccaggatcctcac
L858R	ctcacagcagggtcttctctgtttcagGGCATGAACTACTTGGAGGACCGTCGCTTGGTGCACCGCGACCTGGCAGCCAGGAACGTACTGGTGAAAACACCGCAGCATGTCAAGATCACAGATTTTGGGCGGGCCAAACTGCTGGGTGCGGAAGAGAAAGAATACCATGCAGAAGGAGGCAAAgtaaggaggtggctttaggtcagccagcattttcctgacaccagggaccaggctgccttcccactagctgtattgtttaacacatgcaggggaggatgctctccagacattctgggt
exon 19 deletion (Δ746-750)	TgcggctccacagccccagtgtccctcaccttcggggtgcatcgctggtaacatccacccagatcactgggcagcatgtggcaccatctcacaattgccagttaacgtcttccttctctctctgtcatagGGACTCTGGATCCCAGAAGGTGAGAAAGTTAAAATTCCCGTCGCTATCAAAACATCTCCGAAAGCCAACAAGGAAATCCTCGATgtgagtttctgctttgctgtgtgggggtccatggctctgaacctcaggcccaccttttctcatgtctggcagctgctctgctctagaccctgctcatctccacatcctaaatgttcactttctatgtctttccctttctagctctagtgggtataactccctccccttagagacagcactggcctctcccatgctggtatccaccccaaaa

Supplementary Table 5: Droplet digital PCR conditions used to measure the synthetic constructs. (A) Mastermix composition. (B) PCR cycling conditions

A

Reagent	Final Concentration
2x Supermix for probes (no dUTP)	1x
20x gene specific ddPCR assay	900 nM primers/250 nM probe
Template	1 µl
Water	Adjust to a final volume of 20 µl
Final reaction volume	20 µl

B

Temperature, °C	Time	Ramp Rate	Number of Cycles
95	10 min		1
94	30 secs	2°C/sec	40
55	1 min		
98	10 min		1
4	Infinite	1°C/sec	1

Supplementary Table 6: Clinical characteristics of patients included in the study. See Supplementary_Table_6

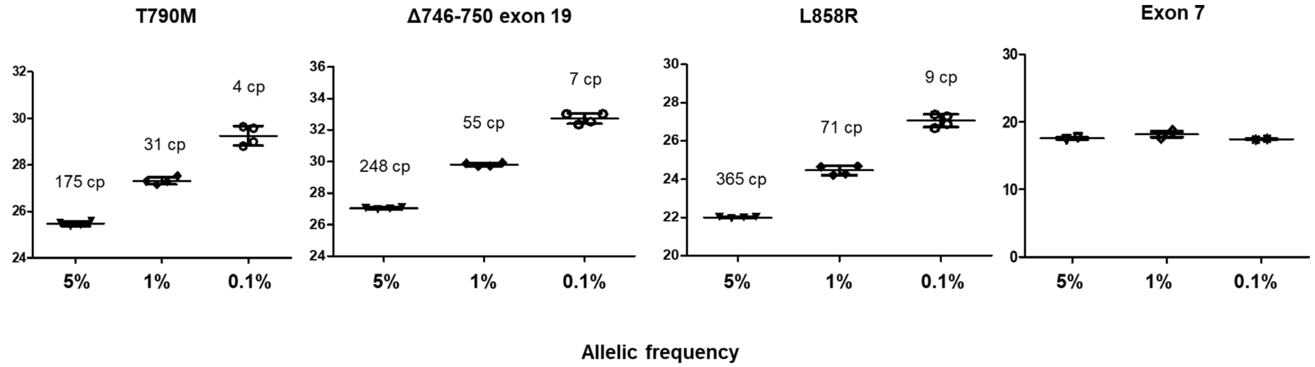

Supplementary Figure 1: Evaluation of assay performance on commercial admixture standards.

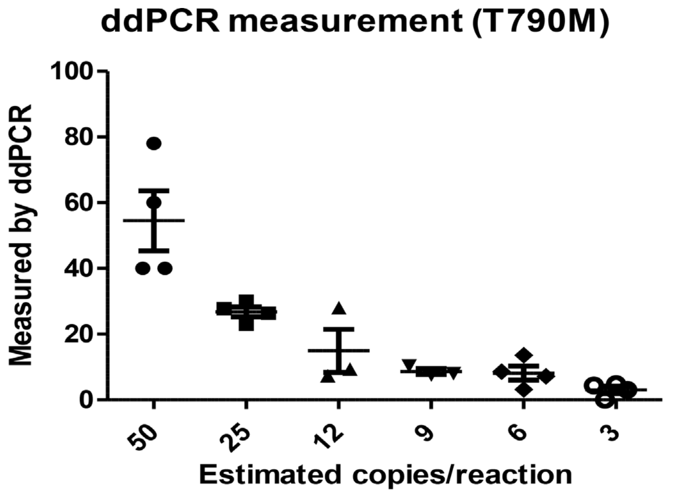

Estimated	Measured ± Stdev
50 copies	54±17.4
25 copies	26.8±2.9
12 copies	14.3±10.2
9 copies	12.5±7.7
6 copies	8.15±4.3
3 copies	2.3±2.7

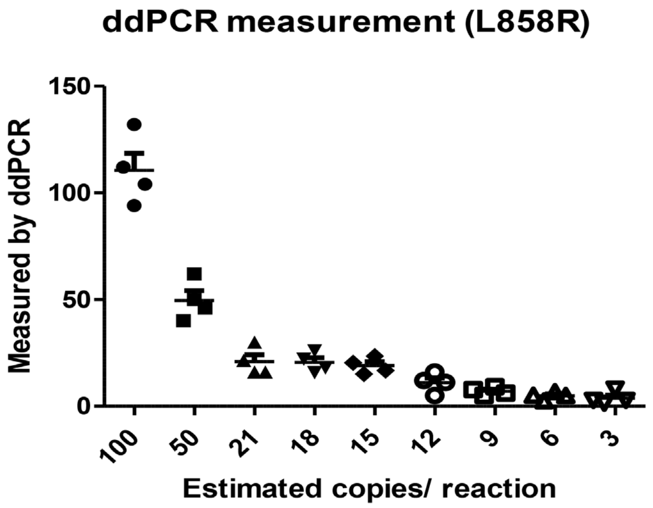

Estimated	Measured ± Stdev
100 copies	110.5±16.1
50 copies	49.5±9.3
21 copies	20.9±6.6
18 copies	20.5±4.5
15 copies	19±3.8
12 copies	11.1±4.5
9 copies	7.1±1.8
6 copies	4.9±1.7
3 copies	3.8±2.9

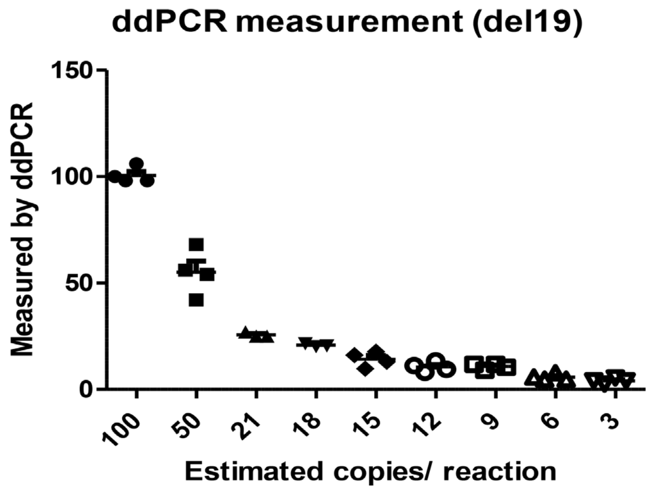

Estimated	Measured ± Stdev
100 copies	100.5±3.8
50 copies	55±10.6
21 copies	24.65±2.2
18 copies	17.05±7.5
15 copies	14.2±3.5
12 copies	10.5±2.3
9 copies	10.75±1.3
6 copies	5.8±1.4
3 copies	4±1.1

Supplementary Figure 2: Droplet digital PCR measurements used for the spike-in experiments in plasma background.
