# Supplementary material for: Exosome-based detection of activating and resistance EGFR mutations from plasma of non-small cell lung cancer patients
Source: Oncotarget. 2019 Apr 23;10(30):2911–20. doi: 10.18632/oncotarget.26885 (PMC6499599; doi:10.18632/oncotarget.26885)
Supplement: Supplementary file 2 [file oncotarget-10-2911-s002.docx]

**Supplementary Table 6.** **Clinical characteristics of patients included in the study**

| **Patient no.** | **Sample type** | **Gender** | **Age*** | **Histology** | **Tumor stage** | **M status** | **T790M tissue status** | **L858R tissue status** | **exon 19 indels tissue status** |
| --- | --- | --- | --- | --- | --- | --- | --- | --- | --- |
| 1 | Healthy | Male | 30 | NA | NA | NA | NA | NA | NA |
| 2 | Healthy | Male | 32 | NA | NA | NA | NA | NA | NA |
| 3 | Healthy | Female | 32 | NA | NA | NA | NA | NA | NA |
| 4 | Healthy | Female | 31 | NA | NA | NA | NA | NA | NA |
| 5 | Healthy | Female | 40 | NA | NA | NA | NA | NA | NA |
| 6 | Healthy | Female | 34 | NA | NA | NA | NA | NA | NA |
| 7 | Healthy | Male | 40 | NA | NA | NA | NA | NA | NA |
| 8 | Healthy | 6 M/6 F | 37.5* | NA | NA | NA | NA | NA | NA |
| 9 | Healthy | 8 M/8 F | 48* | NA | NA | NA | NA | NA | NA |
| 10 | Healthy | 24 M/24 F | 34* | NA | NA | NA | NA | NA | NA |
| 11 | Healthy | 5 M/5 F | 41* | NA | NA | NA | NA | NA | NA |
| 12 | Healthy | 10 M/10 F | 44* | NA | NA | NA | NA | NA | NA |
| 13 | Healthy | 10 M/10 F | 46* | NA | NA | NA | NA | NA | NA |
| 14 | Healthy | 4 Males | 34* | NA | NA | NA | NA | NA | NA |
| 15 | Healthy | 4 Females | 41* | NA | NA | NA | NA | NA | NA |
| 16 | Healthy | 10 M/30 F | 35* | NA | NA | NA | NA | NA | NA |
| 17 | Healthy | 24 M/24 F | 41* | NA | NA | NA | NA | NA | NA |
| 18 | Healthy | Male | 43 | NA | NA | NA | NA | NA | NA |
| 19 | Healthy | Male | 55 | NA | NA | NA | NA | NA | NA |
| 20 | Healthy | Male | 59 | NA | NA | NA | NA | NA | NA |
| 21 | Healthy | Male | 54 | NA | NA | NA | NA | NA | NA |
| 22 | Healthy | Male | 49 | NA | NA | NA | NA | NA | NA |
| 23 | Healthy | Male | 45 | NA | NA | NA | NA | NA | NA |
| 24 | Healthy | Male | 48 | NA | NA | NA | NA | NA | NA |
| 25 | Healthy | Male | 40 | NA | NA | NA | NA | NA | NA |
| 26 | NSCLC | Male | 62 | Squamous or epidermoid | II | T2aN1M0 | Negative | Negative | Negative |
| 27 | NSCLC | Male | 65 | Squamous or epidermoid | Ia | T1bN0M0 | Negative | Negative | Negative |
| 28 | NSCLC | Male | 70 | Squamous or epidermoid | IIa | T2bN0M0 | Negative | Negative | Negative |
| 29 | NSCLC | Male | 75 | Squamous or epidermoid | IIa | T2aN0M0 | Negative | Negative | Negative |
| 30 | NSCLC | Male | 61 | Squamous or epidermoid | Ib | T2aN0M0 | Negative | Negative | Negative |
| 31 | NSCLC | Male | 70 | Squamous or epidermoid | IIIa | T3N1M0 | Negative | Negative | Negative |
| 32 | NSCLC | Male | 36 | Squamous or epidermoid | IIa | T2bN0M0 | Negative | Negative | Negative |
| 33 | NSCLC | Male | 76 | Squamous or epidermoid | I | T1aN0M0 | Negative | Negative | Negative |
| 34 | NSCLC | Male | 61 | Squamous or epidermoid | Ib | T2aN0M0 | Negative | Negative | Negative |
| 35 | NSCLC | Male | 63 | Adenocarcinoma | IIa | T2aN1M0 | Negative | Negative | Negative |

**Supplementary Table 6 (continuation).** Clinical characteristics of patients included in the study

| **Patient no.** | **Sample type** | **Gender** | **Age*** | **Histology** | **Tumor stage** | **M status** | **T790M tissue status** | **L858R tissue status** | **exon 19 indels tissue status** |
| --- | --- | --- | --- | --- | --- | --- | --- | --- | --- |
| 36 | NSCLC | Unknown | Unknown | Unknown | Unknown | Unknown | Negative | Negative | Negative |
| 37 | NSCLC | Unknown | Unknown | Unknown | Unknown | Unknown | Negative | Negative | Negative |
| 38 | NSCLC | Unknown | Unknown | Unknown | Unknown | Unknown | Negative | Negative | Negative |
| 39 | NSCLC | Unknown | Unknown | Unknown | Unknown | Unknown | Negative | Negative | Negative |
| 40 | NSCLC | Unknown | Unknown | Unknown | Unknown | Unknown | Negative | Negative | Negative |
| 41 | NSCLC | Unknown | Unknown | Unknown | Unknown | Unknown | Negative | Negative | Negative |
| 42 | NSCLC | Unknown | Unknown | Unknown | Unknown | Unknown | Negative | Negative | Negative |
| 43 | NSCLC | Unknown | Unknown | Unknown | Unknown | Unknown | Negative | Negative | Negative |
| 44 | NSCLC | Unknown | Unknown | Unknown | Unknown | Unknown | Negative | Negative | Negative |
| 45 | NSCLC | Unknown | Unknown | Unknown | Unknown | Unknown | Negative | Negative | Negative |
| 46 | NSCLC | Unknown | Unknown | Unknown | Unknown | Unknown | Negative | Negative | Negative |
| 47 | NSCLC | Unknown | Unknown | Unknown | Unknown | Unknown | Negative | Negative | Negative |
| 48 | NSCLC | Unknown | Unknown | Unknown | Unknown | Unknown | Negative | Negative | Negative |
| 49 | NSCLC | Unknown | Unknown | Unknown | Unknown | Unknown | Negative | Negative | Negative |
| 50 | NSCLC | Unknown | Unknown | Unknown | Unknown | Unknown | Negative | Negative | Negative |
| 51 | NSCLC | Male | 69 | Adenocarcinoma | IV | M1b | Positive | Positive | Negative |
| 52 | NSCLC | Female | 73 | Adenocarcinoma | IV | M1b | Positive | Negative | Positive |
| 53 | NSCLC | Female | 78 | Adenocarcinoma | IV | M1b | Positive | Negative | Positive |
| 54 | NSCLC | Male | 59 | Adenocarcinoma | IIIB | M1b | Positive | Negative | Positive |
| 55 | NSCLC | Male | 57 | Adenocarcinoma | IV | M1b | Positive | Positive | Negative |
| 56 | NSCLC | Female | 68 | Adenocarcinoma | IV | M1b | Positive | Negative | Positive |
| 57 | NSCLC | Male | 72 | Adenocarcinoma | IV | M1a | Positive | Negative | Positive |
| 58 | NSCLC | Female | 68 | Adenocarcinoma | I | M0 | Positive | Negative | Positive |
| 59 | NSCLC | Female | 67 | Adenocarcinoma | IV | M1a | Positive | Positive | Negative |
| 60 | NSCLC | Male | NA | Adenocarcinoma | IV | M1b | Positive | Positive | Negative |
| 61 | NSCLC | Male | NA | Adenocarcinoma | 0 | M0 | Positive | Negative | Positive |
| 62 | NSCLC | Female | NA | Adenocarcinoma | I | M1b | Positive | Negative | Positive |
| 63 | NSCLC | Female | 65 | Adenocarcinoma | IIIA | M1a | Positive | Positive | Negative |
| 64 | NSCLC | Male | 60 | Adenocarcinoma | IV | M1b | Positive | Negative | Positive |
| 65 | NSCLC | Female | 51 | Adenocarcinoma | IV | M1b | Positive | Negative | Positive |
| 66 | NSCLC | Female | 69 | Adenocarcinoma | IV | M1b | Positive | Negative | Positive |
| 67 | NSCLC | Male | 79 | Adenocarcinoma | IV | M1b | Positive | Negative | Positive |
| 68 | NSCLC | Female | 76 | Adenocarcinoma | IV | M1a | Positive | Negative | Positive |
| 69 | NSCLC | Female | 67 | Adenocarcinoma | IV | M1b | Positive | Negative | Positive |
| 70 | NSCLC | Male | 67 | Adenocarcinoma | IV | M1b | Positive | Negative | Positive |
| 71 | NSCLC | Female | 70 | Adenocarcinoma | IV | M1b | Positive | Positive | Negative |
| 72 | NSCLC | Male | 75 | Adenocarcinoma | IV | M1a | Positive | Positive | Negative |
| 73 | NSCLC | Male | 70 | Adenocarcinoma | IV | M1a | Positive | Negative | Positive |
| 74 | NSCLC | Female | 51 | Adenocarcinoma | IV | M1a | Positive | Negative | Positive |

**Supplementary Table 6 (continuation).** Clinical characteristics of patients included in the study

| **Patient no.** | **Sample type** | **Gender** | **Age*** | **Histology** | **Tumor stage** | **M status** | **T790M tissue status** | **L858R tissue status** | **exon 19 indels tissue status** |
| --- | --- | --- | --- | --- | --- | --- | --- | --- | --- |
| 75 | NSCLC | Female | 60 | Adenocarcinoma | IV | M1a | Positive | Negative | Positive |
| 76 | NSCLC | Female | 59 | Adenocarcinoma | IV | M1b | Positive | Positive | Negative |
| 77 | NSCLC | Male | 81 | Adenocarcinoma | IV | M1b | Positive | Positive | Negative |
| 78 | NSCLC | Male | 75 | Unknown | IV | M1b | Positive | Positive | Negative |
| 79 | NSCLC | Male | 68 | Adenocarcinoma | IV | M1b | Positive | Negative | Positive |
| 80 | NSCLC | Female | 73 | Adenocarcinoma | IV | M1b | Positive | Positive | Negative |
| 81 | NSCLC | Female | 59 | Unknown | IV | M1a | Positive | Positive | Negative |
| 82 | NSCLC | Male | 66 | Adenocarcinoma | IV | M1b | Positive | Positive | Negative |
| 83 | NSCLC | Female | 78 | Adenocarcinoma | II | M0 | Positive | Negative | Positive |
| 84 | NSCLC | Female | 55 | Adenocarcinoma | IV | M1b | Positive | Positive | Negative |
| 85 | NSCLC | Female | 67 | Adenocarcinoma | I | M1b | Positive | Positive | Negative |
| 86 | NSCLC | Male | 58 | Adenocarcinoma | IV | M1b | Positive | Negative | Positive |
| 87 | NSCLC | Female | 66 | Adenocarcinoma | IV | M1b | Positive | Positive | Negative |
| 88 | NSCLC | Male | 76 | Adenocarcinoma | IV | M1b | Positive | Negative | Positive |
| 89 | NSCLC | Female | 65 | Adenocarcinoma | IV | M1a | Positive | Negative | Positive |
| 90 | NSCLC | Female | 92 | Adenocarcinoma | IIIB | M1a | Positive | Negative | Positive |
| 91 | NSCLC | Male | 64 | Adenocarcinoma | IV | M1b | Positive | Negative | Positive |
| 92 | NSCLC | Female | 77 | Adenocarcinoma | IV | M0 | Positive | Negative | Positive |
| 93 | NSCLC | Female | 70 | Adenocarcinoma | IV | M1b | Positive | Negative | Positive |
| 94 | NSCLC | Male | 66 | Unknown | IIIA | M1b | Positive | Negative | Positive |
| 95 | NSCLC | Female | 52 | Adenocarcinoma | IV | M1a | Positive | Negative | Positive |
| 96 | NSCLC | Female | 65 | Adenocarcinoma | IV | M1b | Positive | Negative | Positive |
| 97 | NSCLC | Female | 93 | Adenocarcinoma | IIIA | M0 | Positive | Positive | Negative |
| 98 | NSCLC | Female | 72 | Adenocarcinoma | II | M1a | Positive | Positive | Negative |
| 99 | NSCLC | Female | 66 | Adenocarcinoma | IIIB | M1b | Positive | Negative | Positive |
| 100 | NSCLC | Male | 78 | Adenocarcinoma | IV | M1b | Positive | Negative | Positive |
| 101 | NSCLC | Female | 61 | Adenocarcinoma | IV | M1b | Positive | Negative | Positive |
| 102 | NSCLC | Female | 55 | Adenocarcinoma | IV | M1b | Positive | Negative | Positive |
| 103 | NSCLC | Female | 69 | Adenocarcinoma | IV | M1b | Positive | Positive | Negative |
| 104 | NSCLC | Male | 56 | Adenocarcinoma | IV | M1b | Positive | Negative | Positive |
| 105 | NSCLC | Female | 81 | Adenocarcinoma | IV | M1b | Positive | Negative | Positive |
| 106 | NSCLC | Female | 64 | Adenocarcinoma | IV | M1b | Positive | Negative | Positive |
| 107 | NSCLC | Male | 65 | Adenocarcinoma | IV | M1b | Positive | Negative | Positive |
| 108 | NSCLC | Unknown | Unknown | Unknown | Unknown | Unknown | Positive | Negative | Positive |
| 109 | NSCLC | Unknown | Unknown | Unknown | Unknown | Unknown | Positive | Negative | Positive |
| 110 | NSCLC | Unknown | Unknown | Unknown | Unknown | Unknown | Positive | Negative | Positive |

* For the pooled samples, the number summarized in the table is the average age between all the individual donor’s ages.
